# Heterozygous ABCC6 mutation in a patient with sickle cell disease: Pseudoxanthoma elasticum or pseudoxanthoma elasticum-like phenotype?

**DOI:** 10.1016/j.jdcr.2025.12.025

**Published:** 2025-12-24

**Authors:** José Cuesta Camuñas, Bianca C. Rodríguez Martínez, Fabiola Pabón-González, Melanie Medina-Figueroa, Rafael Martin

**Affiliations:** a Department of Dermatology, University of Puerto Rico, Medical Sciences Campus, San Juan, Puerto Rico; b Department of Pediatrics, University of Puerto Rico, Medical Sciences Campus, San Juan, Puerto Rico

**Keywords:** heterozygous ABCC6 mutation, pseudoxanthoma elasticum, PXE, sickle cell disease

## Introduction

Pseudoxanthoma elasticum (PXE) is a rare autosomal recessive disease characterized by the presence of yellowish cobblestone-like papules and plaques in flexural areas. Other affected organs include the eyes and the cardiovascular system, with significant morbidity and mortality. PXE is caused by a mutation in the ABCC6 gene, which encodes a transmembrane ATP-binding efflux transporter, resulting in ectopic mineralization of connective tissue and degeneration of elastic fibers present in affected organs.^1^

Patients with hemoglobinopathies can present skin lesions very similar to those seen in PXE. Patients with thalassemia intermedia, thalassemia major, and sickle cell disease (SCD) presenting PXE-like cutaneous lesions with characteristic histologic findings have been reported in the literature.^2^ The presence of ABCC6 mutations in these patients has only rarely been investigated. Two patients with SCD and PXE-like lesions with homozygous mutations in ABCC6 (concurrent diagnosis of SCD and PXE) have been identified.^3^^,^^4^ We present a case of a patient with SCD with PXE-like skin changes and a heterozygous mutation for the ABCC6 gene, which to the best of our knowledge, has never been reported.

## Case report

**Fig 1:**
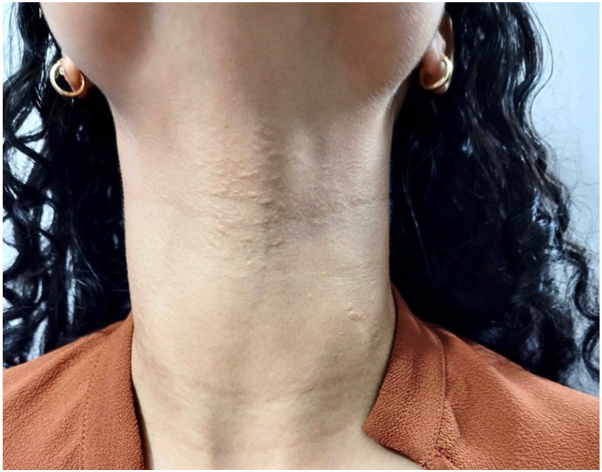
*Yellow papules* coalescing into cobblestoned plaques resembling plucked chicken skin in anterior and posterolateral neck.

**Fig 2:**
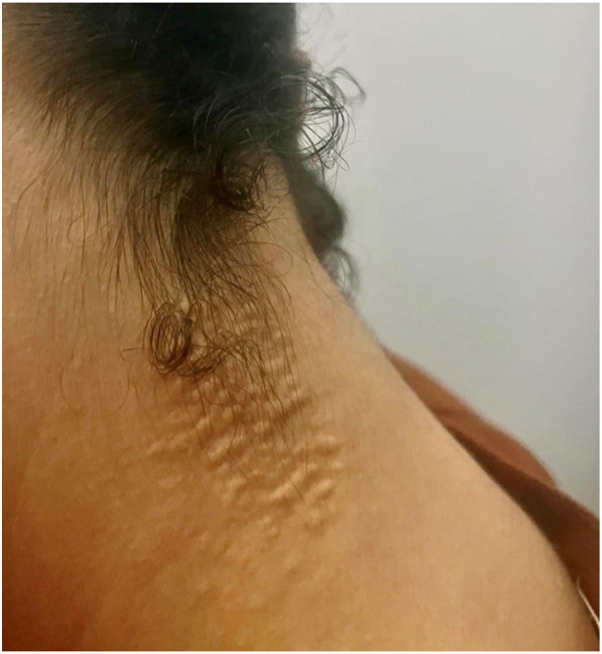
*Yellow papules* coalescing into cobblestoned plaques resembling plucked chicken skin in anterior and posterolateral neck.

A 22-year-old female with a past medical history of SCD, managed with hydroxyurea and folic acid, presented to the dermatology clinic with slowly progressing asymptomatic skin lesions in the neck of 3 years evolution. The skin lesions appeared as yellow papules coalescing into cobblestoned plaques resembling plucked chicken skin in the anterior and posterolateral neck (Fig 1 & Fig 2). The patient had no history of associated fatigue, malaise, shortness of breath, ocular, or gastrointestinal symptoms. Family history was pertinent for sickle cell trait, but no similar skin lesions were previously reported by family members.

**Fig 3:**
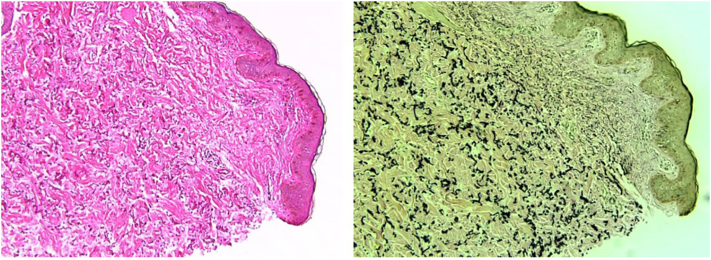
H&E (10×) granular calcification in the dermis.

Laboratory results were remarkable for macrocytic hyperchromic anemia (hemogloblin [Hgb]– 10.1 g/dL, mean corpuscular volume - 125 fL, mean corpuscular hemoglonin - 41.1 pg), expected in the setting of the patient’s condition and medication use. Metabolic, lipid, and coagulation panels were unremarkable. Punch biopsy revealed granular calcification in the dermis. Verhoeff's Van Gieson stain showed small fragmented and curled elastic fibers (Fig 3). Retinal examination by ophthalmology service failed to demonstrate angioid streaks or Peau d'orange changes. Genetic testing revealed a missense mutation in the ABCC6 gene (p.Arg1214Trp), which is considered a pathogenic variant. Clinicopathologic correlation was consistent with a diagnosis of PXE-like skin changes in a patient with SCD who, in addition, is a heterozygous carrier for PXE.

## Discussion

PXE-like changes in patients with hemoglobinopathies may represent a diagnostic conundrum for dermatologists since both patients with SCD and PXE can present with similar cutaneous lesions. The most recent diagnostic criteria revised in 2017 divide findings into major and minor criteria. Characteristic skin manifestations, ocular findings, and 2 pathogenic variants of the ABCC6 gene are considered major criteria, whereas smaller ocular manifestations or a single pathogenic variant are considered minor criteria. A definitive diagnosis of PXE is established by the presence of at least 2 major criteria. A probable diagnosis is defined as 2 major skin or 2 major ocular findings, or presence of 1 major and 1 minor criterion. A possible diagnosis is defined as having 1 major or 1 or minor criteria. If genetic analysis is negative or unavailable, hemoglobinopathies and PXE-like phenotypes are excluded from a definitive diagnosis.^2^ Even though established diagnostic criteria exclude patients with hemoglobinopathies from a definitive diagnosis of PXE, 2 cases of concurrent classic PXE disease confirmed with genetic tests and hemoglobinopathies have been reported in the literature.^3^^,^^4^

We describe a case of a female patient with SCD who presented with yellowish skin papules in the neck clinically and pathologically indistinguishable from cutaneous lesions of PXE. Genetic testing revealed 1 pathogenic mutation of the ABCC6 gene, thus suggesting a probable diagnosis of PXE. However, the diagnostic conundrum in this case is that, according to diagnostic criteria, our patient may not have classic PXE but may have concurrent SCD and possible or probable PXE. Although PXE is typically considered an autosomal recessive condition, our patient's findings raise questions about whether these PXE-like cutaneous findings are secondary to either variable penetrance in a PXE carrier, SCD with PXE-like skin changes (no PXE) or synergistic potentiation of susceptible PXE carriers with SCD.

Previous studies exploring heterozygous carriers for PXE have rarely reported skin involvement. Martin et al reported histologic findings consistent with PXE in heterozygous individuals with no visible skin manifestations, suggesting a phenotypic spectrum of the disease process.^5^ Heterozygous carriers of PXE are commonly considered healthy, although some publications suggest possible increased risk of cardiovascular and/or ophthalmologic complications such as coronary artery disease and vision loss, suggesting the need for active surveillance in these patients.^1^^,^^5^^,^^6^ Furthermore, patients with a heterozygous mutation in the ABCC6 gene are considered to have a probable or possible diagnosis of PXE. Thus, if they are less than 30 years old, they are considered to have a provisional diagnosis and require re-evaluation,^2^ as is the case for our patient, further supporting the need for surveillance and follow up.

Although rare, some patients with hemoglobinopathies and PXE-like cutaneous lesions have been reported to develop a milder “acquired” phenotype of PXE, with symptoms tending to appear in early adulthood.^7^ Available evidence suggests that patients with SCD and PXE-like ophthalmic or cutaneous lesions warrant further evaluation with genetic testing to rule out concomitant PXE, which could have more serious manifestations such as cardiovascular complications which usually appear years after the onset of ocular or cutaneous lesions.^2^

Our patient presented with PXE-like skin changes without ocular or cardiovascular manifestations and a heterozygous mutation of the ABCC6 gene, representing the coexistence of SCD and PXE carrier state. Given the possible risk of eventual development of PXE manifestations, multidisciplinary care was established as recommended in the literature.

Although the exact mechanism of ectopic mineralization in these conditions has not been determined, it results from cumulative and progressive faulty deposition of calcium and phosphate in elastic fibers.^1^ Some studies suggest that oxidative stress secondary to hemolysis in patients with hemoglobinopathies can lead to cumulative damage in connective tissue, hence producing PXE-like cutaneous and ophthalmologic lesions^2^^,^^7^^,^^8^ This further emphasizes the need for long-term follow-up in these patients.

To the best of our knowledge, SCD patients with PXE-like cutaneous lesions heterozygous for the PXE gene have not been previously reported. Thus, our patient may be considered to have both SCD and be a carrier of PXE, suggesting variable penetrance or potentiation of PXE-like lesions in a susceptible individual with a concomitant risk factor such as SCD. Therefore, our patient will require continuous multidisciplinary care including ophthalmologists, cardiologists, and gastroenterologists, to monitor complications along with genetic counseling. Future diagnostic criteria for PXE may consider modifications to include patients with hemoglobinopathies presenting with PXE-like skin changes and PXE heterozygous mutations.

## Conflicts of interest

None disclosed.
